# Impact of early initiation of renal replacement therapy in patients on venoarterial ECMO using target trial emulation with Japanese nationwide data

**DOI:** 10.1038/s41598-025-85109-9

**Published:** 2025-01-07

**Authors:** Toshihiro Kubo, Tomonori Takeuchi, Norihiko Inoue, Augusto Cama-Olivares, Deepak Chandramohan, Ashita J. Tolwani, Keith M. Wille, Kiyohide Fushimi, Javier A. Neyra, Kenji Wakabayashi

**Affiliations:** 1 Department of Intensive Care Medicine, Institute of Science Tokyo, 1-5-45 Yushima, Bunkyo-Ku, Tokyo, 113-8510 Japan; 2 Division of Nephrology, Department of Medicine, University of Alabama at Birmingham, THT 647, 1720 2nd Avenue S, Birmingham, AL 35233 USA; 3 Department of Health Policy and Informatics, Institute of Science Tokyo, 1-5-45 Yushima, Bunkyo-Ku, Tokyo, 113-8510 Japan; 4 Department of Internal Medicine, Brookwood Baptist Health, 833 Princeton Avenue SW, Birmingham, AL 35211 USA; 5 Division of Pulmonary, Allergy and Critical Care Medicine, Department of Medicine, University of Alabama at Birmingham, 1900 University Blvd, Birmingham, AL 35294 USA

**Keywords:** Venoarterial extracorporeal membrane oxygenation, Renal replacement therapy, Target trial emulation, Outcomes research, Continuous renal replacement therapy, Cardiac device therapy

## Abstract

While renal replacement therapy (RRT) allows for precise fluid management as well as addressing electrolyte imbalances and the removal of other necessary compounds, its early initiation has not shown benefit in the general critically ill population. Moreover, the effects of early RRT initiation specifically in patients on venoarterial extracorporeal membrane oxygenation (VA-ECMO) also remain unclear. This retrospective study investigated adult patients who underwent VA-ECMO between April 2018 and March 2022 and used the clone-censor-weight method to emulate a hypothetical target trial and compare two groups: patients who initiated RRT within 2 days of VA-ECMO initiation (Early) and those who did not (Late). The primary outcomes were 28-day and 90-day hospital mortality analyzed by Cox proportional hazards models and the secondary outcome was 90-day RRT dependence by pooled logistic regression models. Inverse probability censoring weights were applied to adjust the models. A total of 2,513 VA-ECMO patients were cloned into both groups. The 28-day and 90-day mortalities were lower in the Early group (HR 0.59 [95% CI 0.53–0.68] and 0.67 [0.61–0.75]). However, the early group experienced greater RRT dependence at 90 days than the late group (OR 2.58 [1.94–3.46]). In conclusion, early initiation of RRT (within 2 days of VA-ECMO) was associated with lower hospital mortality but with a higher likelihood of 90-day RRT dependence in adult patients on VA-ECMO.

**Supplementary Information:**

The online version contains supplementary material available at 10.1038/s41598-025-85109-9.

## Introduction

Extracorporeal membrane oxygenation (ECMO) is a lifesaving intervention and venoarterial ECMO (VA-ECMO) is used for patients with severe cardiovascular failure. Patients requiring ECMO are critically ill, frequently presenting with multiple organ failure, and are at high risk of developing acute kidney injury (AKI), which occurs in up to 85% of ECMO patients^1–4^. AKI is a recognized risk factor for mortality in patients receiving ECMO^4,5^, and renal replacement therapy (RRT) is required in 40 to 50% of these patients^5,6^.

Patients with VA-ECMO may develop AKI due to severe cardiogenic shock as well as other factors, including hypotension, insufficient renal perfusion, renal congestion due to fluid overload or congestive heart failure, the presence of inflammatory mediators, ischemia-reperfusion injury, and/or hemolysis^7–10^. Patients on VA-ECMO also face risks of transfusion overload because of volume resuscitation and coagulation disorders, with renal congestion from excessive transfusion during shock potentially exacerbating the AKI^11^. In fact, fluid management is a common indication of RRT initiation in patients on ECMO. According to a previous worldwide survey, the management (43%) or prevention (16%) of fluid overload were the main reasons for initiating RRT during ECMO^12^. Furthermore, a retrospective cohort study has shown that fluid accumulation during ECMO is associated with worse prognosis, suggesting that RRT to remove excess fluid may improve clinical outcomes^13^.

Previous studies examining the optimal timing of RRT initiation in general ICU populations have suggested that early initiation may not improve mortality^14–16^. However, patients on ECMO were either underrepresented or not adequately reported in these studies, and clinical outcomes of early versus delayed initiation of RRT during ECMO have not been specifically explored. The Target Trial Emulation is a novel causal inference method in observational studies that addresses not only confounding bias but also immortal time and selection bias^17^. Dynamic clinical parameters can be incorporated into the modeling of treatment strategies to account for their influence^18^. Based on the above, the goal of our study was to determine whether early RRT initiation during VA-ECMO improves mortality and other clinical outcomes using the novel Target Trial Emulation method.

## Methods

### Study design and data source

This is a retrospective analysis of the Diagnosis Procedure Combination (DPC) database as a comprehensive and nationwide health claims data source to conduct a hypothetical target trial. The DPC database is an administrative health claims database in Japan, involving more than 1,700 hospitals, most of which are acute care hospitals with 92% (244/266) coverage of tertiary medical institutions in Japan^19^. The database includes patient demographic data, diagnostic information based on ICD-10 codes, patient dispositions, treatments, surgeries, medications, and the use of medical devices and supplies. From this database, we extracted hospital admission information from April 2018 to March 2022 for our study patients, and acquired outpatient and inpatient information up to five years prior to each admission as pre-admission data. This study was conducted in accordance with the Declaration of Helsinki and approved by the Ethics Review Board of Institute of Science Tokyo (M2000-788), which also waived the requirement for informed consent from patients due to the anonymization of the data.

### Study participants

Our study targeted adult patients aged 18 and over who were admitted to the ICU and used VA-ECMO during the specified period. Since information on the specific type of ECMO support was not available in the DPC database during the study period, we identified VA-ECMO cases based on ICD codes, following a previous DPC study with modifications^20^, and included patients diagnosed with cardiovascular disease, pulmonary embolism, hypothermia, poisoning, or trauma (a list of ICD codes is available in Supplementary Table 2). Cases where ECMO charges were considered as temporary use of cardiopulmonary bypass during cardiac surgery were excluded. We also excluded patients who did not have ECMO initiated on the day of or the day after ICU admission, patients who used RRT before the initiation of ECMO, patients with a diagnosis of end-stage kidney disease (ESKD) either during the current hospital stay or in outpatient/inpatient data from the past five years (Supplementary Table 2). Additionally, we excluded patients diagnosed with viral pneumonia to preclude patients on venovenous ECMO (Supplementary Table 2). A design diagram visualizing the above is available in the Supplementary Materials (Supplementary Fig. 1)^21^.

### Treatment strategies (intervention and comparison)

We defined the two treatment strategies compared in the hypothetical target trial based on the timing of initiating RRT. The patient cohort that initiated RRT on the day of or the day after starting VA-ECMO was classified as the Early group, and the cohort that did not was classified as the Late group. The implementation of RRT was defined as the presence of recorded charges both for the RRT procedure itself and for the use of dialysis fluid or replacement fluid.

### Study outcomes

The primary outcomes were hospital mortality at 28 days and 90 days from the initiation of VA-ECMO (Supplementary Fig. 2). The secondary outcome was RRT dependence at 90 days from the initiation of VA-ECMO. RRT dependence was defined as follows: (1) In patients hospitalized for more than 90 days, if RRT is undergone at 90 days; (2) For periods of 90 days or less, if RRT is undergone in the 3 days prior to discharge or death. Additionally, death was considered as a competing risk for RRT dependence (Supplementary Fig. 2).

### Covariates

We included factors that could affect treatment selection and outcomes as model covariates, including: age, sex, smoking status (never smoker vs. current/past smoker), admission due to cardiovascular disease, presence of obesity (defined as a body mass index of ≥ 30 or not), Charlson comorbidity index (CCI)^22,23^, presence of chronic kidney disease (CKD) based on ICD-10 codes, emergency admission status, ICU type (1 to 4, according to Japanese health system), each component of the SOFA score at the initiation of ECMO, concurrent use of intra-aortic balloon pump (IABP), use of Impella, use of ventricular assist device (VAD), administration of antibiotics, implementation of targeted temperature management, use of vasopressors, use of inotropes, volume of intravenous fluid administration (adjusted for days and body weight, and categorized by tertile), execution of cardiac surgery, execution of aortic aneurysm surgery, execution of percutaneous coronary intervention (PCI), use of loop diuretics, and exposure to nephrotoxins. Detailed definitions for each covariate are described in the Supplementary Table 3. Of note, there were missing values for smoking status, height, weight, and each component of SOFA score, which were imputed using Multivariate Imputation by Chained Equations (MICE)^24^. The missing rates for these variables are available in Supplementary Table 5.

### Statistical analysis

We employed a novel causal inference concept known as the Target Trial Emulation using the clone-censor-weight method to address confounding, selection, and immortal time bias in observational studies^17,25^. Initially, cloning involved copying the entire cohort data to create two identical cohorts. One was designated as the arm to assign to the Early group, and the other to the Late group. Next, we processed artificial censoring for patients who deviated from the protocols of their respective treatment strategies (Supplementary Fig. 3). Specifically, in the Early group, if RRT was not initiated by the day following the start of ECMO, it was considered as a deviation from the emulated protocol. Similarly, patients in the Late group who started RRT on the day of or the day after starting ECMO were also considered deviations and thus censored. While cloning ensures both groups have the same baseline characteristics, the introduction of artificial censoring presents a potential source of bias as treatment strategies were not randomly assigned. To address this, we utilized inverse probability of censoring weighting (IPCW), which is essential for adjusting the bias introduced by artificial censoring. This adjustment makes the comparison between the groups fair, enhancing the validity of our analysis despite the non-random assignment of treatment strategies. IPCW was calculated as the probability of remaining uncensored at each event, employing a Cox regression model that included the aforementioned covariates. Utilizing this weighting to analyze mortality as the primary outcome, we applied the Cox proportional hazards model. We calculated the adjusted survival from this model and plotted the adjusted survival curve up to 90 days from the initiation of ECMO. For secondary outcomes of RRT dependence, we used pooled logistic regression, considering death as a competing risk event. Estimation of hazard ratios (HR) or odds ratios (OR) with their 95% confidence intervals (CI) was achieved through nonparametric bootstrapping, with the process repeated 1,000 times. Furthermore, we conducted analyses for primary and secondary outcomes in the crude cohort without cloning. Similar to the analysis in the Target Trial Emulation, univariate and multivariate Cox proportional hazards models for mortality and logistic regression for RRT dependence were used in the crude cohort analysis. Covariates in these multivariate models were the same ones used in the IPCW for the Target Trial Emulation.

As a sensitivity analysis, we conducted the same analysis on the primary outcomes for a cohort excluding patients with CKD, encompassing analyses in both the crude cohort and the Target Trial Emulation. This is because we incorporated CKD as a covariate in the models without considering the severity levels of the condition, which could lead to misclassification of ESKD or the underestimation of patients at high risk for RRT. Additionally, as a supplemental analysis, we conducted the same analysis on the secondary outcome (RRT dependence), limiting the cohort to survivors only.

## Results

### Characteristics of the crude cohort

**Fig. 1:**
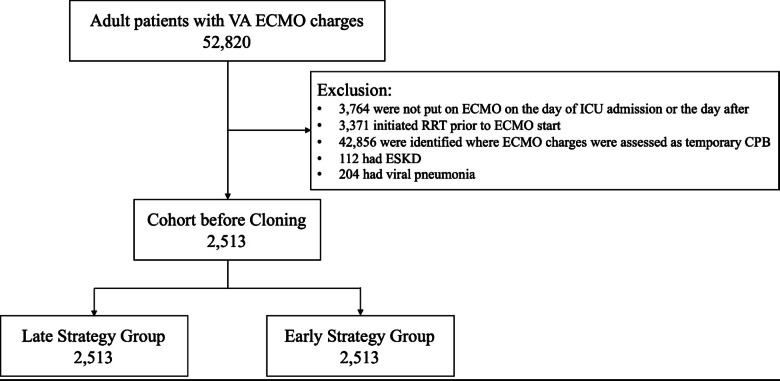
Flow diagram of the study population. *VA-ECMO* venoarterial, *ICU* intensive care unit, *RRT* renal replacement therapy, *CPB* cardiopulmonary bypass, *ESKD* end-stage kidney disease.

Among 52,820 adult patients who underwent VA-ECMO, 2,513 were selected for analysis based on pre-defined eligibility criteria (Fig. 1). Across the entire cohort, the median age was 69 years (interquartile range [IQR] 56–77), 35.4% were female, and 98.3% were admitted for cardiovascular disease (Table 1). The prevalence of CKD was 5.4%, and the median CCI was 2 (IQR 0–2). The median SOFA score and its renal component were 10 (IQR 8–12) and 0 (IQR 0–2), respectively. 516 patients (20.5%) started RRT within 2 days of ECMO initiation, while 1,997 (79.5%) did not. A total of 1,560 patients (62.0%) did not undergo RRT during hospitalization. The overall mortality rate of the cohort was 61.7%, and the median duration of VA-ECMO was 2 days (IQR 2–4) (Supplementary Table 6).

**Table 1:** Baseline characteristics for patients who underwent VA-ECMO.

	Overall cohort (*n* = 2,513)	Received early RRT (*n* = 516)	Did not receive early RRT (*n* = 1997)
Demographics and comorbidities			
Age (years)	69 [56, 77]	67 [54, 77]	69 [56, 77]
Sex (female)	890 (35.4)	165 (32.0)	725 (36.3)
BMI (kg/m^2^)	23.5 [20.9, 26.2]	24.0 [21.1, 27.0]	23.3 [20.9, 26.0]
Obesity (BMI ≧ 30)	193 (7.7)	48 (9.3)	145 (7.3)
Past or current smoker	1391 (55.4)	322 (62.4)	1069 (53.5)
Chronic kidney disease	135 (5.4)	58 (11.2)	77 (3.9)
Charlson comorbidity index	2 [0, 2]	2 [0, 2]	1 [0, 2]
Baseline characteristics			
Unscheduled admission	1872 (74.5)	367 (71.1)	1505 (75.4)
Reason for hospitalization			
Cardiovascular disease	2470 (98.3)	505 (97.9)	1965 (98.4)
Trauma	64 (2.5)	11 (2.1)	53 (2.7)
Hypothermia	36 (1.4)	8 (1.6)	28 (1.4)
Poisoning	13 (0.5)	4 (0.8)	9 (0.5)
SOFA score at ECMO initiation			
Total	10 [8, 12]	11 [9, 13]	10 [8, 12]
Respiration	2 [1, 3]	2 [1, 3]	2 [1, 3]
Coagulation	1 [0, 2]	1 [0, 2]	1 [0, 2]
Liver	0 [0, 1]	0 [0, 1]	0 [0, 1]
Cardiovascular	2 [1, 4]	3 [2, 4]	2 [1, 4]
Central nerve system	4 [2, 4]	3 [2, 4]	4 [2, 4]
Renal	0 [0, 1]	1 [0, 2]	0 [0, 1]
Procedures and treatments at or before group assignment			
Procedures			
Cardiac surgery	2461 (97.9)	508 (98.4)	1953 (97.8)
Surgery for aneurysm	245 (9.7)	54 (10.5)	191 (9.6)
Percutaneous coronary intervention	538 (21.4)	100 (19.4)	438 (21.9)
Device use			
IABP	244 (9.7)	55 (10.7)	189 (9.5)
Impella	98 (3.9)	27 (5.2)	71 (3.6)
VAD	19 (0.8)	5 (1.0)	14 (0.7)
Target temperature management	115 (4.6)	23 (4.5)	92 (4.6)
Other treatments			
Diuretics	1310 (52.1)	325 (63.0)	985 (49.3)
Loop diuretics	880 (35.0)	229 (44.4)	651 (32.6)
Vasopressors	2155 (85.8)	473 (91.7)	1682 (84.2)
Inotropes	1313 (52.2)	328 (63.6)	985 (49.3)
Antibiotics	1808 (71.9)	438 (84.9)	1370 (68.6)
Sedatives	2012 (80.1)	465 (90.1)	1547 (77.5)
Opioids	1821 (72.5)	433 (83.9)	1388 (69.5)
Exposure to nephrotoxin	631 (25.1)	168 (32.6)	463 (23.2)
Intravenous fluid volume (mL/day/kg)	56.0 [33.3, 91.2]	62.7 [34.8, 101.5]	54.9 [33.1, 86.9]
Blood transfusion	1886 (75.0)	429 (83.1)	1457 (73.0)

Categorical variables are presented as n (%), and numerical variables as median [IQR].

The detailed definitions of each variable are available in Supplementary Tables 2 and 3.

*BMI* body mass index, *SOFA score* sequential organ failure assessment score, *VA-ECMO* venoarterial extracorporeal membrane oxygenation, *IABP* intra-aortic balloon pump, *VAD* Ventricular assist devices.

### Hospital mortality

**Fig. 2:**
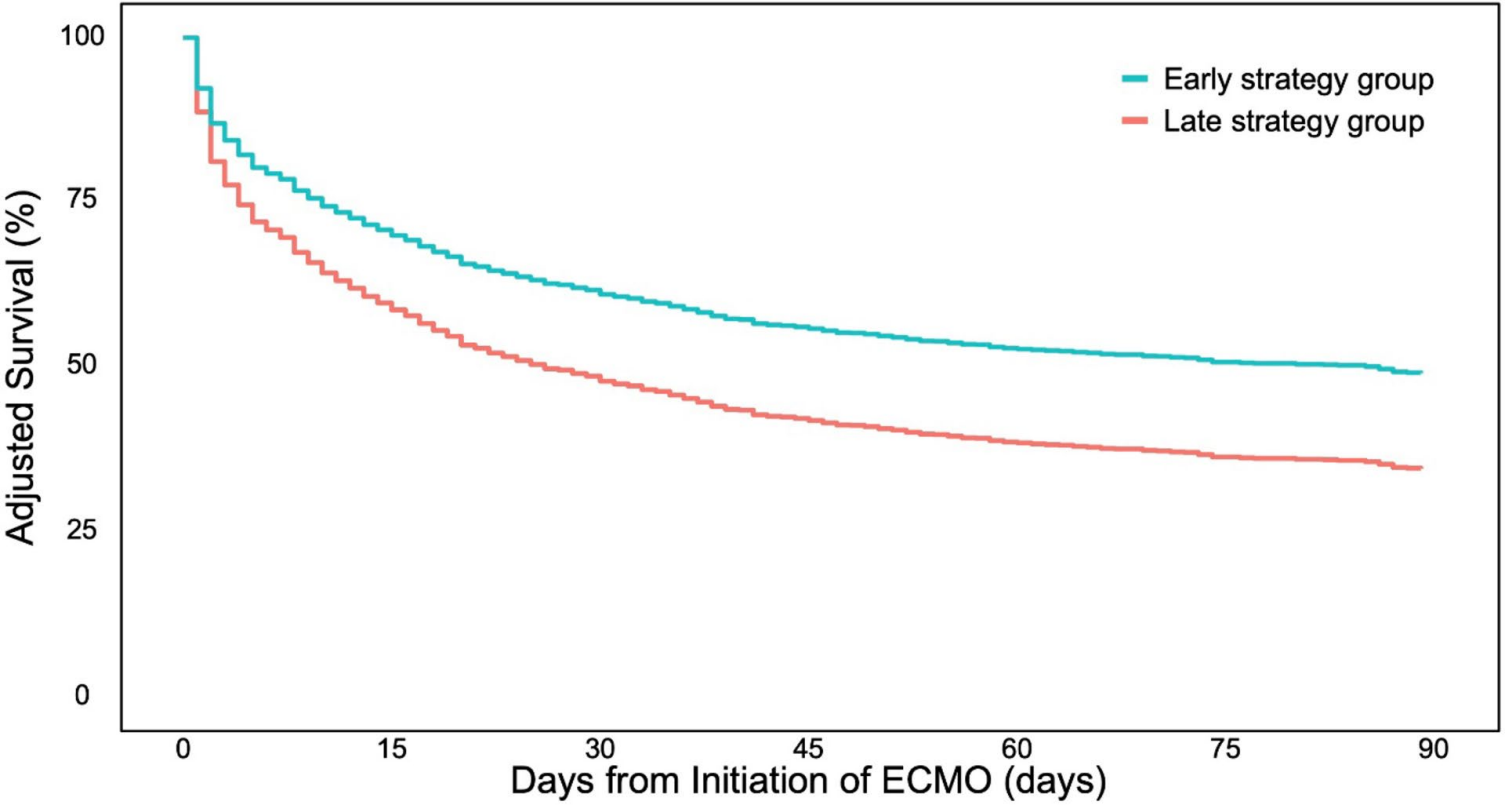
Adjusted survival curve in patients on VA-ECMO stratified by RRT strategy. *VA-ECMO* venoarterial extracorporeal membrane oxygenation. The Y-axis displays the adjusted survival based on the Cox proportional hazards model weighted by IPCW obtained in the Target Trial Emulation. The X-axis represents the number of days from ECMO initiation.

In both the unadjusted models and the models adjusted for covariates of the crude cohort, as well as in the models in the Target Trial Emulation, a reduction in risk for hospital mortality was observed with the Early vs. Late strategy (Fig. 2; Table 2). In the Target Trial Emulation, the HR for 28-day mortality was 0.59 (95% CI 0.53–0.68), and for 90-day mortality, the HR was 0.67 (95% CI 0.61–0.75). The results from the sensitivity analysis using the cohort excluding CKD patients were consistent in all models except for the model adjusted for covariates in the crude cohort for 90-day mortality (HR 0.88, 95% CI 0.76–1.01) (Supplementary Table 7). In the sensitivity analysis, the HR in the Target Trial Emulation models in the sensitivity analysis was 0.58 for 28-day mortality (95% CI 0.52–0.67) and 0.65 for 90-day mortality (95% CI 0.58–0.74).

**Table 2:** Mortality risk in the early strategy group compared to the late strategy group.

	HR	95% CI
28-day mortality		
Model 1	0.77	0.67–0.89
Model 2	0.83	0.71–0.96
Model 3	0.59	0.53–0.68
90-day mortality		
Model 1	0.83	0.73–0.95
Model 2	0.86	0.75–0.99
Model 3	0.67	0.61–0.75

Model 1 is a univariate Cox proportional hazards model in the crude cohort, Model 2 is a multivariate Cox proportional hazards model in the crude cohort, and Model 3 is a Cox proportional hazards model weighted by IPCW in the Target Trial Emulation. Covariates included in Model 2 and IPCW for Mode 3 are age, sex, smoking status, obesity, chronic kidney disease, Charlson comorbidity index, cardiovascular disease, unscheduled admission, Japanese ICU category, each component of the SOFA score, use of IABP, Impella, and VAD, implementation of target temperature management, use of antibiotics, vasopressors, inotropes, and loop diuretics, volume of intravenous fluids, cardiac surgery, aneurysm surgery, percutaneous coronary intervention, and exposure to nephrotoxins.

*HR* hazard ratio, *CI* confidence interval.

### RRT dependence

For 90-day RRT dependence, both models using the crude cohort and those employing the Target Trial Emulation consistently showed increased odds in the Early group (Table 3). The OR for 90-day RRT dependence in the Early group in the Target Trial Emulation model was 2.58 (95% CI 1.04–3.46). In the analysis of survivors only, increased odds in the Early group were observed in all models (Supplementary Table 8). In the Target Trial Emulation model for survivors only, the OR was 3.77 (95% CI 1.66–9.86).

**Table 3:** RRT dependence in the early strategy group compared to the late strategy group.

	OR	95% CI
90-day RRT dependence		
Model 1	5.86	4.62–7.46
Model 2	5.21	3.99–6.80
Model 3	2.58	1.94–3.46

Model 1 is a univariate logistic model in the crude cohort, Model 2 is a multivariate logistic regression model in the crude cohort, and Model 3 is a pooled logistic regression model weighted by IPCW in the Target Trial Emulation. Covariates included in Model 2 and IPCW for Mode 3 are age, sex, smoking status, obesity, chronic kidney disease, Charlson comorbidity index, cardiovascular disease, unscheduled admission, Japanese ICU category, each component of the SOFA score, use of IABP, Impella, and VAD, implementation of target temperature management, use of antibiotics, vasopressors, inotropes, and loop diuretics, volume of intravenous fluids, cardiac surgery, aneurysm surgery, percutaneous coronary intervention, and exposure to nephrotoxins.

*OR* odds ratio, *CI* confidence interval.

## Discussion

We conducted a retrospective analysis of the Japanese nationwide health claims data to investigate the effects of early RRT initiation on mortality risk and RRT dependence among patients on VA-ECMO. We employed a novel causal inference method known as the Target Trial Emulation, which effectively addresses not only confounding and selection biases but also the immortal time bias^17,18^. Using this method, we found that early initiation of RRT in adult VA-ECMO patients reduced the risk of hospital mortality at both 28 and 90 days, with consistent risk reduction observed in both conventional analyses and those with the Target Trial Emulation. However, for 90-day RRT dependence, the risk was consistently higher with early initiation of RRT.

Numerous studies have explored the timing of RRT initiation in overall critical care populations, with multiple Randomized Controlled Trials (RCTs) failing to show a mortality benefit from early RRT initiation strategies^14–16^. However, these studies mostly excluded patients with high disease severity or did not specify ECMO usage. As a result, the effects on the most severe cohort of patients who are most likely to benefit from meticulous fluid management were removed or diluted in the analyses. In line with this, our study demonstrated a potential mortality benefit from the early RRT initiation strategy in ECMO patients, highlighting that trials involving heterogeneous ICU populations may not represent all ICU patients.

To date, few studies have explored the optimal timing for RRT initiation in ECMO patients, and none are as large-scale as this study. A retrospective cohort study in Korea involving 296 patients, including 210 cases of VA-ECMO and 86 cases of Venovenous ECMO, reported no significant difference in 30-day mortality between ECMO patients who started RRT within 72 h of ECMO initiation and those who did not^26^. Similarly, a single-center pilot RCT in China, which compared an early initiation of RRT within 12 h of starting ECMO (*n* = 21) with standard care (*n* = 20), found no significant difference in 30-day mortality. In these studies, immortal bias in the late group complicates the interpretation, and the Target Trial Emulation used in our study effectively demonstrated the mortality benefit of early RRT initiation that was not proven in the previous study^27^.

Although this study lacks detailed information on how fluid management through RRT was conducted, the observed reduction in mortality risk with early RRT initiation may suggest that early RRT may break the vicious cycle consisting of circulatory failure, venous congestion including renal congestion, renal impairment, and fluid overload in VA-ECMO patients^28^. Previous studies have reported that VA-ECMO patients have a higher risk of developing AKI compared to non-ECMO patients, and it is generally known that AKI increases mortality, and brings more challenges in fluid management^1,29,30^. The Extracorporeal Life Support Organization guideline recommends initiating RRT for ECMO patients if fluid overload by AKI is unresponsive to diuretics, or AKI impedes the recovery from circulatory/respiratory failure^31^. Our study indicates that proactive use of RRT from the early days of VA-ECMO management improves patient outcomes.

It is intriguing to find that RRT dependence at 90 days was greater in the Early group in this study. This result is consistent with the STARRT AKI trial, but not with a past meta-analysis published before the STARRT AKI trial^14,32^. One possible reason is the hemodynamic changes associated with RRT. Intermittent hemodialysis and continuous RRT have been suggested to reduce renal perfusion^33^, induce myocardial stunning^34^, and alter hemodynamics^35^, which may subsequently contribute to decreased urine output and impaired kidney function^36,37^. It is also uncertain whether these changes occur in the severely critical condition of VA-ECMO. In addition to hemodynamic changes, inflammation due to exposure to RRT may also contribute. Intensive RRT use has been associated with inflammatory markers and apoptosis markers, such as TNFR1, and this inflammatory environment has been associated with poor renal prognosis^38–41^. This potential impact may have been more pronounced in the Early group, which had longer exposure to RRT.

However, we must acknowledge the limitations to our work. As a retrospective analysis of data recorded in the database, we could not account for unknown or unmeasured confounders, including the detailed indications for VA-ECMO. Additionally, owing to the nature of Japanese health claims data, the method of selecting the target population of VA-ECMO patients based on ICD codes, while used in previous studies^20^, may have caused misclassification and selection bias. Moreover, detailed clinical information, such as laboratory data and fluid balance, was unavailable, making it challenging to consider specific parameters related to the indication for RRT as well as the assessment of AKI stages. Furthermore, the lack of detailed data on CKD stage and pre-admission baseline kidney function means that the results cannot be generalized to CKD patients. Finally, the absence of information on not initiating or discontinuing RRT due to futility before death may have introduced potential indication bias and affected the results. However, the strength of our study lies in enhanced validity of our findings due to utilization of nationwide large-scale data and minimization biases through the application of the Target Trial Emulation approach, including cloning and adjustment using IPCW with 28 covariates, as well as conducting sensitivity analyses.

## Conclusions

Initiating RRT within 2 days of starting VA-ECMO was associated with improved hospital mortality but also with an increased risk of 90-day RRT dependence. Further investigation is needed to explore causal inferences using a wider variety of data sources and to conduct evaluations of long-term outcomes, impacts on patient quality of life, and cost-effectiveness.

## Electronic supplementary material

Below is the link to the electronic supplementary material.

Supplementary Material 1

## Data Availability

Data will be shared upon reasonable request to the corresponding author.

## Abbreviations

AKI: Acute kidney injury
BMI: Body mass index
CCI: Charlson comorbidity index
CI: Confidence interval
CKD: Chronic kidney disease
DPC: Diagnosis procedure combination
ECMO: Extracorporeal membrane oxygenation
ESKD: End stage kidney disease
HR: Hazard ratio
IABP: Intra-aortic balloon pump
ICU: Intensive care unit
IPCW: Inverse probability censoring weighting
MICE: Multivariate imputation by chained equations
OR: Odds ratio
PCI: Percutaneous coronary intervention
RRT: Renal replacement therapy
SOFA: Sequential organ failure assessment
VA-ECMO: Venoarterial extracorporeal membrane oxygenation
VAD: Ventricular assist device
