# Supplementary material for: Impact of early initiation of renal replacement therapy in patients on venoarterial ECMO using target trial emulation with Japanese nationwide data
Source: Sci Rep. 2025 Jan 7;15:1074. doi: 10.1038/s41598-025-85109-9 (PMC11707199; doi:10.1038/s41598-025-85109-9)
Supplement: Supplementary file 1 — Supplementary Material 1 [file 41598_2025_85109_MOESM1_ESM.docx]

**Supplementary Materials for**

**Impact of Early Initiation of Renal Replacement Therapy in Patients on Venoarterial ECMO Using Target Trial Emulation with Japanese Nationwide Data**

Toshihiro Kubo*, Tomonori Takeuchi*, Norihiko Inoue, Augusto Cama-Olivares,
Deepak Chandramohan, Ashita J. Tolwani, Keith M. Wille, Kiyohide Fushimi, Javier A. Neyra, Kenji Wakabayashi†

**Supplementary Table 1: STROBE Checklist**

**Supplementary Figure 1: Design Diagram**

**Supplementary Table 2: ICD-10 Codes Definitions for Diagnostic Variables**

**Supplementary Table 3: Definitions and Classifications for Non-Diagnostic Variables**

**Supplementary Table 4: Summary of Criteria for ICU Category in Japan**

**Supplementary Table 5: The number of missing values imputed by Multivariate Imputation by Chained Equations (MICE)**

**Supplementary Figure 2: Definitions for Outcome Measures**

**Supplementary Figure 3: Visualization of Artificial Censoring for the Target Trial Emulation**

**Supplementary Table 6: Clinical outcomes for patients who underwent VA-ECMO**

**Supplementary Table 7: Sensitivity Analysis Excluding Patients with Chronic Kidney Disease for Mortality Risk in the Early Strategy Group Compared to the Late Strategy Group**

**Supplementary Table 8: Sensitivity Analysis in Survivors Only for RRT dependence in the Early Strategy Group Compared to the Late Strategy Group**

* Co-First Authors

† Corresponding Author

**Supplementary Table 1: STROBE Checklist**

|  | Item No | Recommendation | Page No |
| --- | --- | --- | --- |
| **Title and abstract** | 1 | (*a*) Indicate the study’s design with a commonly used term in the title or the abstract | 1-2 |
|  |  | (*b*) Provide in the abstract an informative and balanced summary of what was done and what was found | 2 |
| Introduction | | | |
| Background/rationale | 2 | Explain the scientific background and rationale for the investigation being reported | 3-4 |
| Objectives | 3 | State specific objectives, including any prespecified hypotheses | 4 |
| Methods | | | |
| Study design | 4 | Present key elements of study design early in the paper | 4 |
| Setting | 5 | Describe the setting, locations, and relevant dates, including periods of recruitment, exposure, follow-up, and data collection | 4-5 |
| Participants | 6 | (*a*) Give the eligibility criteria, and the sources and methods of selection of participants. Describe methods of follow-up | 5-6 |
|  |  | (*b*) For matched studies, give matching criteria and number of exposed and unexposed | - |
| Variables | 7 | Clearly define all outcomes, exposures, predictors, potential confounders, and effect modifiers. Give diagnostic criteria, if applicable | 5-7, S Table 2-4, S Fig 2-3 |
| Data sources/ measurement | 8 | For each variable of interest, give sources of data and details of methods of assessment (measurement). Describe comparability of assessment methods if there is more than one group | 5-8 |
| Bias | 9 | Describe any efforts to address potential sources of bias | 7-8 |
| Study size | 10 | Explain how the study size was arrived at | - |
| Quantitative variables | 11 | Explain how quantitative variables were handled in the analyses. If applicable, describe which groupings were chosen and why | 6 |
| Statistical methods | 12 | (*a*) Describe all statistical methods, including those used to control for confounding | 7-8 |
|  |  | (*b*) Describe any methods used to examine subgroups and interactions | 8 |
|  |  | (*c*) Explain how missing data were addressed | 7, S Table 5 |
|  |  | (*d*) If applicable, explain how loss to follow-up was addressed | 7 and S Fig 2-3 |
|  |  | (*e*) Describe any sensitivity analyses | 8 |
| Results | | |  |
| Participants | 13* | (a) Report numbers of individuals at each stage of study—eg numbers potentially eligible, examined for eligibility, confirmed eligible, included in the study, completing follow-up, and analysed | 8-9 and Fig 1 |
|  |  | (b) Give reasons for non-participation at each stage |  |
|  |  | (c) Consider use of a flow diagram |  |
| Descriptive data | 14* | (a) Give characteristics of study participants (eg demographic, clinical, social) and information on exposures and potential confounders | 8-9 |
|  |  | (b) Indicate number of participants with missing data for each variable of interest |  |
|  |  | (c) Summarise follow-up time (eg, average and total amount) |  |
| Outcome data | 15* | Report numbers of outcome events or summary measures over time | 9-10, S Table 6 |

| Main results | 16 | (*a*) Give unadjusted estimates and, if applicable, confounder-adjusted estimates and their precision (eg, 95% confidence interval). Make clear which confounders were adjusted for and why they were included | 9-10, Table 2-3 |
| --- | --- | --- | --- |
|  |  | (*b*) Report category boundaries when continuous variables were categorized | - |
|  |  | (*c*) If relevant, consider translating estimates of relative risk into absolute risk for a meaningful time period | - |
| Other analyses | 17 | Report other analyses done—eg analyses of subgroups and interactions, and sensitivity analyses | 9-10, S Table 7-8 |
| Discussion | | | |
| Key results | 18 | Summarise key results with reference to study objectives | 10 |
| Limitations | 19 | Discuss limitations of the study, taking into account sources of potential bias or imprecision. Discuss both direction and magnitude of any potential bias | 12 |
| Interpretation | 20 | Give a cautious overall interpretation of results considering objectives, limitations, multiplicity of analyses, results from similar studies, and other relevant evidence | 10-12 |
| Generalisability | 21 | Discuss the generalisability (external validity) of the study results | 10-12 |
| Other information | | | |
| Funding | 22 | Give the source of funding and the role of the funders for the present study and, if applicable, for the original study on which the present article is based | 19 |

**Supplementary Figure 1: Design Diagram**

The blue items (light blue and navy blue) represent the variables used for exclusion and their respective windows.

The red items (orange and red) represent the adjustment covariates and their respective windows. The green item represents the outcome measurement period.

Reference:

Schneeweiss S, Rassen JA, Brown JS, Rothman KJ, Happe L, Arlett P, et al. Graphical Depiction of Longitudinal Study Designs in Health Care Databases. Ann Intern Med. 2019;170:398–406.

**Supplementary Table 2: ICD-10 Codes Definitions for Diagnostic Variables**

| **Condition** | **ICD-10 Code** |
| --- | --- |
| Cardiovascular disease | I01, I05 - I09, I20 - I25, I30 - I52 |
| Pulmonary embolism | I26 |
| Hypothermia | T68 |
| Poisoning | T36 - T65 |
| Trauma | S00 - S99, T00 - T14 |
| Viral pneumonia | U071, U072, J09 - J12, J17, B012, B052, B068, B250 |
| Chronic kidney disease | N18.1 - N18.5, N18.9 |
| End-stage kidney disease | N18.5 |

Diagnostic information in Japanese Diagnosis Procedure Combination database is entered based on the 2013 version of the ICD-10 codes.

The ICD-10 codes included in the calculation algorithm for the Charlson Comorbidity Index are based on the definitions provided by Quan et al (*).

Reference:

* Quan, H. et al. Coding algorithms for defining comorbidities in ICD-9-CM and ICD-10 administrative data. *Med. Care* **43**, 1130-1139 (2005).

**Supplementary Table 3: Definitions and Classifications for Non-Diagnostic Variables**

| **Variable** | **Description** |
| --- | --- |
| Age (years) | Age at hospital admission |
| Sex | Male or Female |
| BMI (kg/m2) | Calculated from weight and height at admission |
| Obesity | BMI of 30 kg/m^2^ or above |
| Smoking status | Never smoker if Smoking index is 0 at admission, past or current smoker if greater |
| Chronic kidney disease | Identified by referring to outpatient and inpatient data for 5 years prior to admission, based on ICD-10 codes in Supplementary Table 2 |
| Charlson comorbidity index | Calculated based on concurrent diseases at admission using ICD-10 codes. The ICD-10 codes included in the calculation algorithm for the Charlson Comorbidity Index are based on the definitions provided by Quan et al (*). The model incorporates this as a score categorized into 3 levels: 0, 1 or 2, and 3 or above |
| Emergency admission status | Whether the admission was scheduled or unscheduled |
| ICU category in Japan | In Japan, the "Intensive Care Management Fee" varies by institution and is determined by the ICU category (Type 1 to 4). This categorization is based on factors including physician, nursing staff, and clinical technician criteria as well as the facility and equipment provisions. For details, refer to Supplementary Table 4. |
| Reason for hospitalization | Identified based on ICD-10 codes, the diagnoses leading to hospitalization are categorized into diseases related to VA-ECMO, including cardiovascular disease, pulmonary embolism, hypothermia, poisoning, and trauma. Definitions for these categories are available in Supplementary Table 2. |
| SOFA score | The SOFA score components entered by medical staff on the day of ECMO initiation |
| Cardiac surgery | Identified whether surgeries linked to the surgical codes provided by the Federation of Social Insurance Committees of Surgical Associations indicating Cardiovascular sites were performed prior to ECMO initiation during hospitalization |
| Surgery for aneurysm | Identified whether surgeries with codes indicating aortic aneurysm were performed prior to ECMO initiation during hospitalization |
| Percutaneous coronary intervention | Identified whether procedures with codes indicating percutaneous coronary intervention were performed prior to ECMO initiation during hospitalization |
| IABP | Identified if in use at the time of ECMO initiation, based on the calculation of procedure and material costs |
| Impella | Identified if in use at the time of ECMO initiation, based on the calculation of procedure and material costs |
| VAD | Identified if in use at the time of ECMO initiation, based on the calculation of procedure and material costs |
| Target temperature management | Identified if in use at the time of ECMO initiation, based on the calculation of procedure and material costs |
| Diuretics | Identified based on order information if thiazide diuretics, loop diuretics, potassium-sparing diuretics (anti-aldosterone), osmotic diuretics, vasopressin receptor antagonists, and carbonic anhydrase inhibitors were used from hospital admission to ECMO initiation. The models incorporated only loop diuretics. |
| Vasopressors | Identified if drugs such as epinephrine, norepinephrine, dopamine, vasopressin, or phenylephrine were being used at the time of ECMO initiation |
| Inotropes | Identified if drugs such as dobutamine or milrinone were being used at the time of ECMO initiation |
| Antibiotics | Identified if antiviral, antibacterial, antifungal, or antiprotozoal agents were being used at the time of ECMO initiation, based on order information |
| Sedatives | Identified if propofol, midazolam, dexmedetomidine, or ketamine were being used at the time of ECMO initiation, based on order information |
| Opioids | Identified if a combination of morphine, fentanyl, or remifentanil was being used at the time of ECMO initiation, based on order information |
| Exposure to nephrotoxin | Identified based on order information if angiotensin-converting enzyme inhibitors, angiotensin II receptor blockers, aminoglycosides, or nonsteroidal anti-inflammatory drugs were used from hospital admission to ECMO initiation |
| Intravenous fluid volume (mL/day/kg) | Identified intravenous medication orders within one week prior to ECMO initiation and standardized the total volume by body weight and days (mL/kg/day). The model incorporated categories divided into tertiles |
| Blood transfusion | Identified whether any blood transfusion products were used within one week prior to ECMO initiation |

Reference:

* Quan, H. et al. Coding algorithms for defining comorbidities in ICD-9-CM and ICD-10 administrative data. *Med. Care* **43**, 1130-1139 (2005).

**Supplementary Table 4: Summary of Criteria for ICU Category in Japan**

| **ICU Category** | **Physician Criteria** | **Nursing Criteria** | **Clinical Technologist Criteria** | **Facility Criteria** |
| --- | --- | --- | --- | --- |
| Type 1 | At least 2 dedicated physicians with 5+ years of ICU experience must always be on-site | Nurses with 5+ years of ICU experience; completed 600 hours of specific training; at least 20 hours per week on-site | Dedicated clinical engineering technologist always on-site | ≥ 20 m² per bed |
| Type 2 | Meets Type 1 criteria plus dedicated physicians for extensive burns | Meets Type 1 criteria | Meets Type 1 criteria | ≥ 20 m² per bed; treatment room suitable for extensive burns |
| Type 3 | At least one dedicated physician must always be on-site; ICU experience not specified | Not specified | Not specified | ≥ 15 m² per bed |
| Type 4 | Meets Type 3 criteria plus dedicated physicians for extensive burns | Not specified | Not specified | ≥ 15 m² per bed; treatment room suitable for extensive burns |

**Supplementary Table 5: The number of missing values imputed by Multivariate Imputation by Chained Equations**

| **Variable** | | **n (%)** |
| --- | --- | --- |
| Smoking index | | 602 (24.0) |
| Height | | 325 (12.9) |
| Weight | | 300 (11.9) |
| SOFA score | |  |
|  | Respiratory system | 608 (24.2) |
|  | Coagulation | 557 (22.2) |
|  | Liver | 589 (23.4) |
|  | Cardiovascular system | 564 (22.4) |
|  | Central nerve system | 587 (23.4) |
|  | Renal function | 556 (22.1) |

**Supplementary Figure 2: Definitions for Outcome Measures**

**2 - A: Definition for 28-day (90-day) mortality**
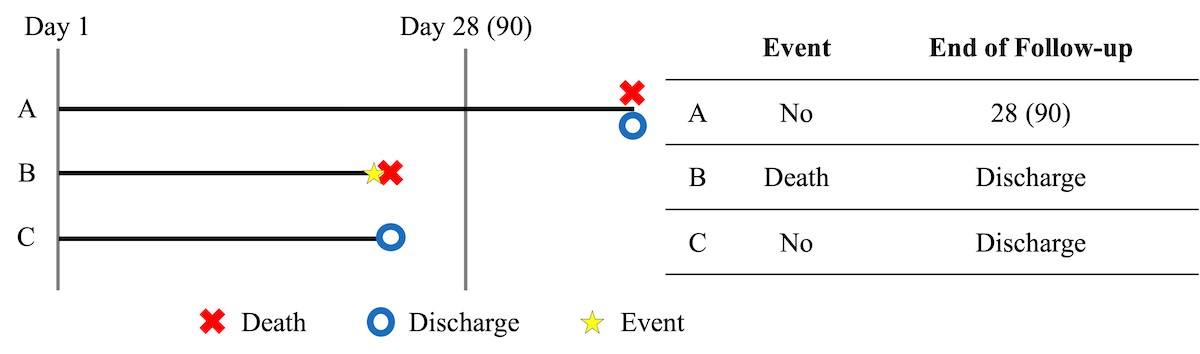


In the Target Trial Emulation, in addition to the above, artificial censoring was performed for the calculation of inverse probability censoring weights.

**2 - B: Definition for 90-day RRT dependence**


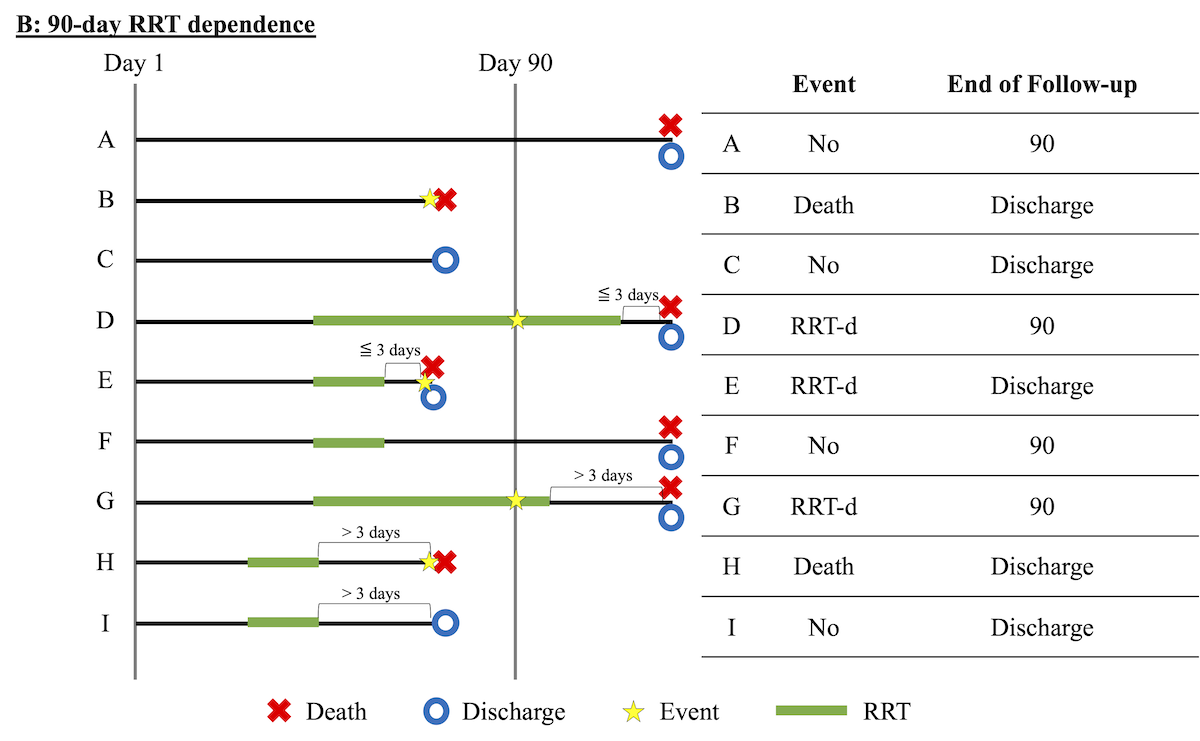
RRT-d, renal replacement therapy dependence.

In the Target Trial Emulation, in addition to the above, artificial censoring was performed for the calculation of inverse probability censoring weights.

**Supplementary Figure 3: Visualization of Artificial Censoring for the Target Trial Emulation**


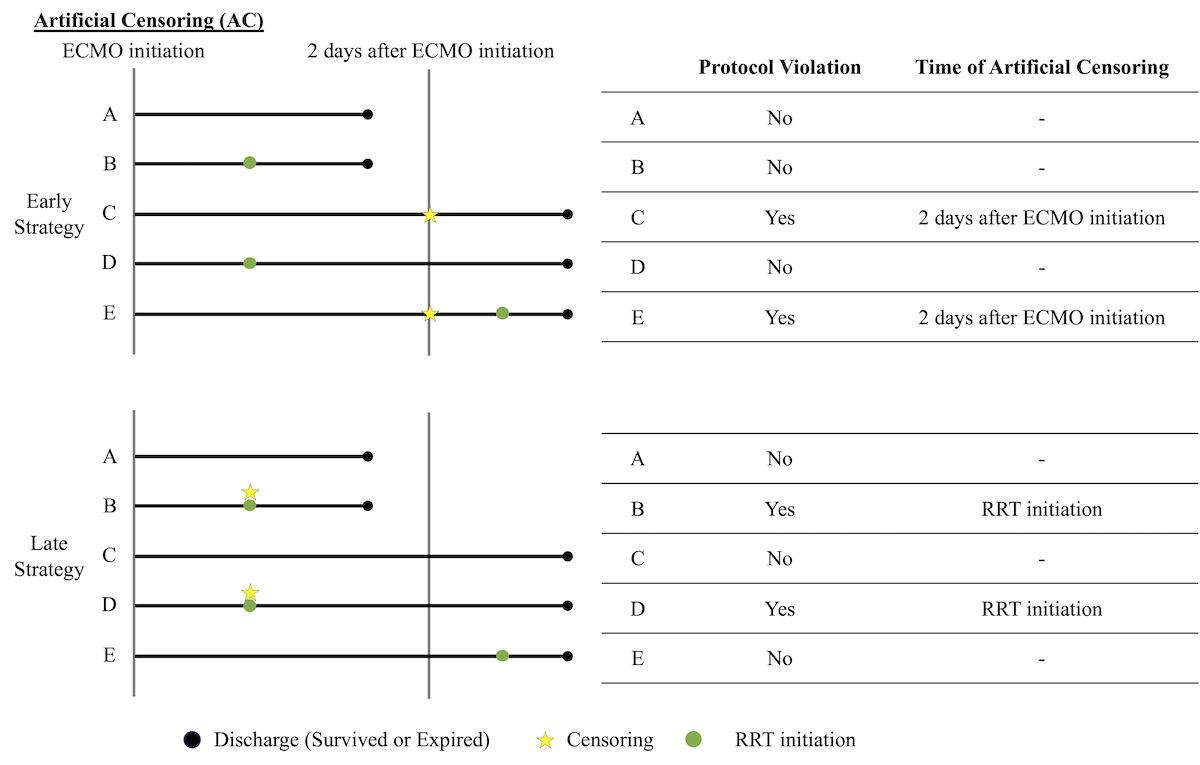


In Target Trial Emulation, to address selection bias in two strategy cohorts with duplicated identical patients, weighting correction is performed using inverse probability censoring weighting (IPCW). The artificial censoring handled by IPCW was implemented as shown in the figure above. The following is a supplementary explanation:

**Early Strategy Group**

- If a patient is discharged within two days following ECMO initiation without undergoing RRT, it does not deviate from the early strategy protocol (A).
- If RRT is initiated within two days after ECMO initiation, it is considered adherence to the protocol (B, D).
- If no RRT is performed within two days after ECMO initiation, and the patient survives beyond this period, it is a deviation from the protocol, and artificial censoring is applied at that time (C, E).

**Late Strategy Group**

- If a patient is discharged within two days following ECMO initiation without undergoing RRT, it does not deviate from the late strategy protocol (A).
- If RRT is initiated within two days after ECMO initiation, it is a deviation from the protocol, and artificial censoring is applied at the time RRT is started (B, D).
- If no RRT is performed within two days after ECMO initiation, and the patient survives beyond this period, it is considered adherence to the protocol (C, E).

**Supplementary Table 6: Clinical outcomes for patients who underwent VA-ECMO**

|  | **Patients on VA-ECMO**  **(n =2,513)** | **Patient who received Early RRT**  **(n =516)** | **Patient who did not receive Early RRT**  **(n =1,997)** |
| --- | --- | --- | --- |
| Hospital mortality | 1550 (61.7) | 327 (63.4) | 1223 (61.2) |
| RRT dependence at discharge | 511 (20.3) | 290 (56.2) | 221 (11.1) |
| Length of hospital stay (days) | 18 [2, 49] | 23 [7, 60] | 17 [2, 46] |
| Length of ICU stay (days) | 8 [2, 14] | 11 [5, 14] | 7 [2, 13] |
| Duration of VA-ECMO (days) | 2 [2, 4] | 3 [2, 6] | 2 [2, 4] |
| Duration of mechanical ventilation (days) | 5 [1, 15] | 9 [3, 21] | 4 [1, 14] |
| Duration of RRT (days) | 0 [0, 4] | 8 [2, 22] | 0 [0, 0] |
| ICU free days at day 28 (days) | 0 [0, 16] | 14 [0, 15] | 0 [0, 16] |
| VA-ECMO free days at day 28 (days) | 0 [0, 25] | 0 [0, 25] | 0 [0, 25] |
| MV free days at day 28 (days) | 0 [0, 16] | 0 [0, 13] | 0 [0, 17] |
| RRT free days at day 28 (days) | 0 [0, 28] | 0 [0, 13] | 0 [0, 28] |

VA-ECMO, venoarterial extracorporeal membrane oxygenation; RRT, renal replacement therapy; ICU, intensive care unit; MV, mechanical ventilation.

Categorical variables are presented as n (%), and numerical variables as median [IQR].

Free-days outcomes were defined as the number of days within a 28-day period from ECMO initiation during which the patient did not require each resource. For patients who died within this 28-day timeframe, the count of free days was set to zero.

**Supplementary Table 7: Sensitivity Analysis Excluding Patients with Chronic Kidney Disease for Mortality Risk in the Early Strategy Group Compared to the Late Strategy Group**

|  | | HR | 95% CI |
| --- | --- | --- | --- |
| 28-day mortality | | | |
|  | Model 1 | 0.79 | 0.68 - 0.91 |
|  | Model 2 | 0.85 | 0.73 - 0.99 |
|  | Model 3 | 0.58 | 0.52 - 0.67 |
| 90-day mortality | | | |
|  | Model 1 | 0.83 | 0.73 - 0.95 |
|  | Model 2 | 0.88 | 0.76 – 1.01 |
|  | Model 3 | 0.65 | 0.58 - 0.74 |

HR, hazard ratio; CI, confidence interval.

Model 1 is a univariate Cox proportional hazards model in the crude cohort, Model 2 is a multivariate Cox proportional hazards model in the crude cohort, and Model 3 is a Cox proportional hazards model weighted by IPCW in the Target Trial Emulation. Covariates included in Model 2 and IPCW for Mode 3 are age, sex, smoking status, obesity, Charlson comorbidity index, cardiovascular disease, unscheduled admission, Japanese ICU category, each component of the SOFA score, use of IABP, Impella, and VAD, implementation of target temperature management, use of antibiotics, vasopressors, inotropes, and loop diuretics, volume of intravenous fluids, cardiac surgery, aneurysm surgery, percutaneous coronary intervention, and exposure to nephrotoxins.

**Supplementary Table 8: Sensitivity Analysis in Survivors Only for RRT dependence in the Early Strategy Group Compared to the Late Strategy Group**

|  | | OR | 95% CI |
| --- | --- | --- | --- |
| 90-day RRT dependence | | | |
|  | Model 1 | 7.01 | 3.58 - 13.72 |
|  | Model 2 | 4.07 | 3.99 - 9.37 |
|  | Model 3 | 3.77 | 1.66 - 9.86 |

OR, odds ratio; CI, confidence interval; RRT, renal replacement therapy.

Model 1 is a univariate logistic model in the crude cohort, Model 2 is a multivariate logistic regression model in the crude cohort, and Model 3 is a pooled logistic regression model weighted by IPCW in the Target Trial Emulation. Covariates included in Model 2 and IPCW for Mode 3 are age, sex, smoking status, obesity, chronic kidney disease, Charlson comorbidity index, cardiovascular disease, unscheduled admission, Japanese ICU category, each component of the SOFA score, use of IABP, Impella, and VAD, implementation of target temperature management, use of antibiotics, vasopressors, inotropes, and loop diuretics, volume of intravenous fluids, cardiac surgery, aneurysm surgery, percutaneous coronary intervention, and exposure to nephrotoxins.
